# Supplementary material for: Prognostic Implications of MRI Melanin Quantification and Cytogenetic Abnormalities in Liver Metastases of Uveal Melanoma
Source: Cancers (Basel). 2021 May 31;13(11):2728. doi: 10.3390/cancers13112728 (PMC8198670; doi:10.3390/cancers13112728)
Supplement: Supplementary file 1 [file cancers-13-02728-s001.zip › cancers-1201253-supplementary.pdf]

cancers

MDPI

Supplementary Material: Prognostic Implications of MRI Melanin Quantification and Cytogenetic Abnormalities in Liver Metastases of Uveal Melanoma

Toulsie Ramtohul, Khadija Ait Rais, Sophie Gardrat, Raymond Barnhill, Sergio Román-Román, Nathalie Cassoux, Manuel Rodrigues, Pascale Mariani, Leanne De Koning, Gaëlle Pierron and Vincent Servois

Text S1: Description of the genetic analysis

Tumor samples were collected in a volume of 10 mL of Hank's balanced salt solution, at room temperature. They were centrifuged at 2000 RPM, decanted then we got back the pellets. They were immediately snap frozen in liquid nitrogen before being stored at -80°C. Then DNA was extracted from cells after 2 h with proteinase K, and 1hour with RNAse A, by a phenol/chloroform and Phase Lock Gel Light (Eppendorf, Hamburg, Germany) procedure. Quantity and quality were estimated in 2 steps:

- A Nanodrop spectrophotometer has been used to assess gDNA purity. For an optimal labeling yield, samples should have an A260/A280≥1.8 and A260/A230≥1.9.
- A Qubit dsDNA BR Assay Kit has been used to measure the double-stranded DNA concentration.

A Whole Genome Amplification can be performed on samples with a too low concentration (10 ng < Concentration < 700 ng), then purified on a QIAamp DNA Mini Kit (QIAGEN, Hilden, North Rhine-Westphalia, Germany). For each sample having a quality and quantity sufficient, 700 to 1000 ng of tumor DNA and reference DNA were labeled, purified and cohybridized in equal quantity to the NimbleGen Arrays or Agilent Microarrays (Roche NimbleGen, Basel, Basel-Stadt, Switzerland), during 12 to 24 h. Human Reference DNA Male and Female were extracted from human blood for those who have passed on a NimbleGen support, unlike those who passed on an Agilent support for which references were provided in the Agilent Kit (Roche NimbleGen, Basel, Basel-Stadt, Switzerland). Arrays were washed and scanned according to their technology. For NimbleGen, images were acquired on a GenePix 4000B scanner with GenePix V.6.6 Software (Molecular Devices, San Jose, CA, USA), and data extracted using the NimbleScan V.2.5 Software. Files produced by Nimblescan V.2.5 Software were then analyzed on SignalMap V.1.9. For Agilent, images were acquired on a SureScan Microarray Scanner (Agilent Technologies, Santa Clara, California, USA) using using CytoScan Software V.2.7, then analysed on CytoGenomics Software V.3.0.2.11. The quality of the technique was evaluated depending on the profile's dynamic, the sex mismatch, and the quality of the dispersion of points.

Text S2: Description of the immunohistochemical assessment of pigmentation of liver metastases

Immunohistochemical analysis of melanin content for each liver metastasis was based on the examination of representative hematoxylin, eosin, saffron-stained sections and utilizing a method developed by R. Barnhill in 2017 involving the recording of (1) the average intensity of cytoplasmic melanin in all melanoma cells in each metastasis with five-point scale (0—absent, 1—faint, 2—moderate, 3—high, 4—very high [1], (2) the overall percentage of the metastasis containing melanin, and (3) the melanin index, which was derived from the (1) intensity of melanin × (2) percentage melanin (%) = melanin index (scale 0 to 400)) [2]. In particular, melanin intensity in melanoma cells was assessed at magnifications 20× and 40× and scored as: 0, (absent) if no pigment was discernible even at high magnification (40×); 1, (faint) pigmentation barely visible at low power. At high power, melanocytes showed faint diffuse melanin or a few pigment granules. 2, (moderate) pigmentation visible at low power with translucent cytoplasm that is significantly

lighter than the hematoxylin-stained nuclei; 3, (high) melanin content easily visible at low magnification with the cytoplasmic pigmentation reaching an intensity approximating that of the nucleus; 4, (very high) cytoplasm strikingly melaninized obscuring nuclei. Melanophages were not evaluated.

Table S1. Metastatic uveal melanoma MRI parameters protocol.

Parameter	T1-Weighted Imaging	T2-Weighted Imaging	DW Imaging	Contrast-Enhanced Imaging
Sequence	Dual gradient echo	Fast spin echo	Echo planar	Gradient echo with three-dimensional acquisition
Fat suppression	No (in phase-out phase)	Yes (spair, spectral)	Yes (spair, spectral)	Yes (quick fat sat, chemical shift selective)
Respiratory triggered	No	Yes	Yes	No
Acquisition time (mn)	0.44	4 to 5 mn	4 to 5 mn	0.24
Repetition time (ms)	101	~1300	~1400	5.75
Echo time (ms)	2.38–4.76	90	69	2.7
Flip angle (degree)	70	138	90	10
Parallel imaging factor	-	-	2	2
Number of signals aquired	1	2	2	1
Field of view (mm)	320 × 300 (93.8%)	320 × 295 (92%)	350 × 262 (75%)	350 × 262 (75%)
Matrix	256 × 230 (90%)	512 × 307 (60%)	192 × 173 (90%)	384 × 307 (80%)
Section thickness (mm)	6	6	6	3.5
Intersection gap (mm)	1.8	1.2	1.8	0.7
Other	Voxel size 1.4 × 1.3 × 6	Voxel size 1 × 0.6 × 6	Voxel size 2 × 1.8 × 6 Echo planar imaging factor: 130 b-values: 0; 50; 300; 600	Voxel size 1.1 × 0.9 × 3.5

Table S2. Univariate Cox proportional hazards analysis of overall survival.

Covariate	Reference	Hazard Ratio (95% CI)	*P*-value
Age (years)	≤60	1.5 (0.8–2.7)	0.22
	>60		
Gender	Female	1.3 (0.7–2.3)	0.42
	Male		
Largest basal diameter (mm)	≤15	0.7 (0.4–1.3)	0.31
	>15		
Ciliar involvement	No	0.5 (0.2–1.0)	0.065
	Yes		
Retinal detachement	No	0.7 (0.4–1.3)	0.28
	Yes		
Ocular tumor treatment	Enucleation	1.3 (0.7–2.4)	0.44
	Proton beam/I-125 plaque		
Disease-free interval (months)	≤24	3.2 (1.7–6.0)	<0.001
	>24		
Size of liver metastases (mm)	≤20	1.6 (0.8–3.3)	0.19
	>20		
Number of liver metastases	≤4	0.7 (0.3–1.3)	0.23
	>4		
Miliary disease	No	0.6 (0.3–1.2)	0.14
	Yes		
Loss of BAP1	Yes	1.1 (0.6–1.9)	0.82
	No/undone		
Genetic classification	High risk	2.5 (1.3–4.6)	0.005
	Low/intermediate risk		
Quality of liver resection	R0	0.2 (0.1–0.5)	<0.001
	R2		
Number of therapeutic lines	≤2	0.7 (0.4–1.3)	0.23
	>2		
Systemic treatment	Yes	4.3 (1.5–12.3)	0.006
	No		

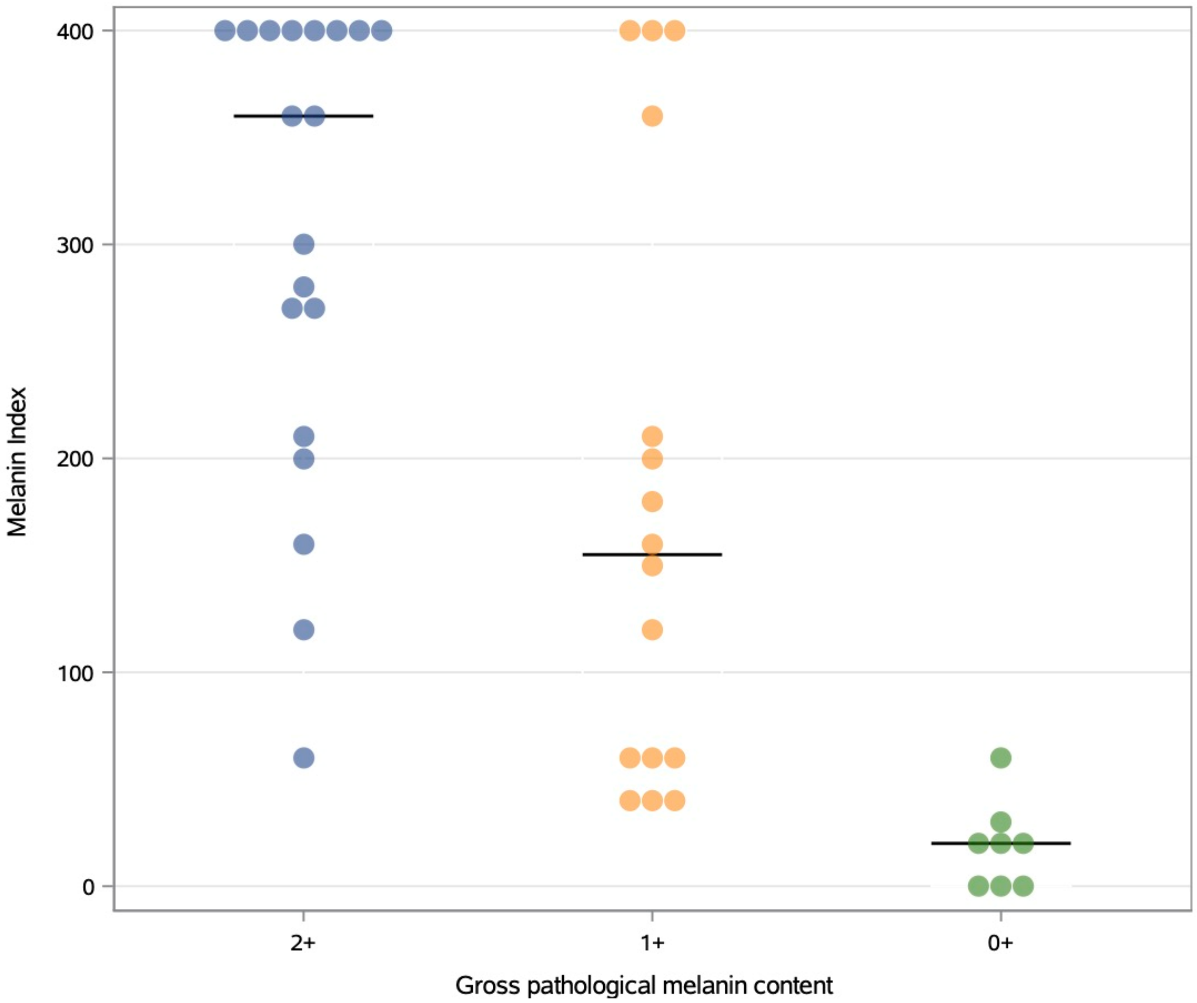

Figure S1. Association between gross pathological and immunophenotypical melanin assessment. Horizontal lines indicate median value. Melanin pathological quantification was assessed visually and graded as follows: 0+ (hypopigmented), 1+ (mixed pigmented), 2+ (strongly pigmented). Melanin index (scale 0 to 400) was derived from the intensity of cytoplasmic melanin and the percentage of melanin. Pearson correlation $r = 0.7$, $p < 0.001$.

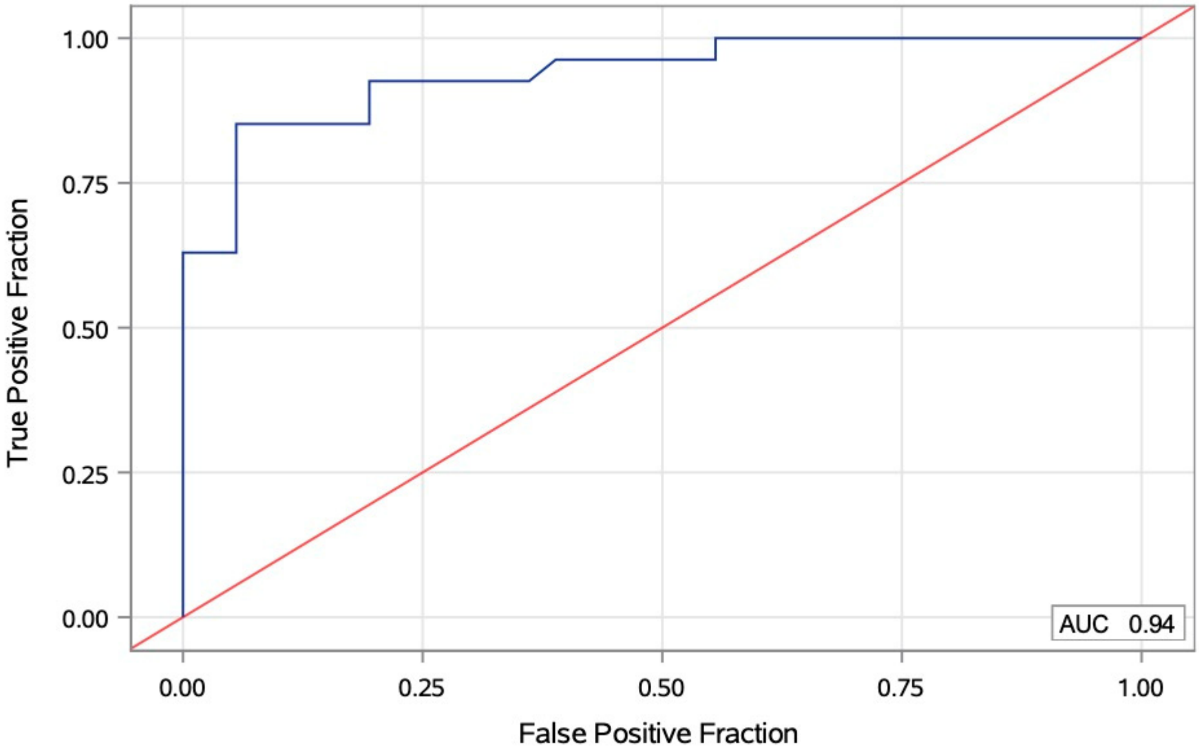

Figure S2. Operating receiving curve of MRI melanin quantification and gross pathological melanin content.

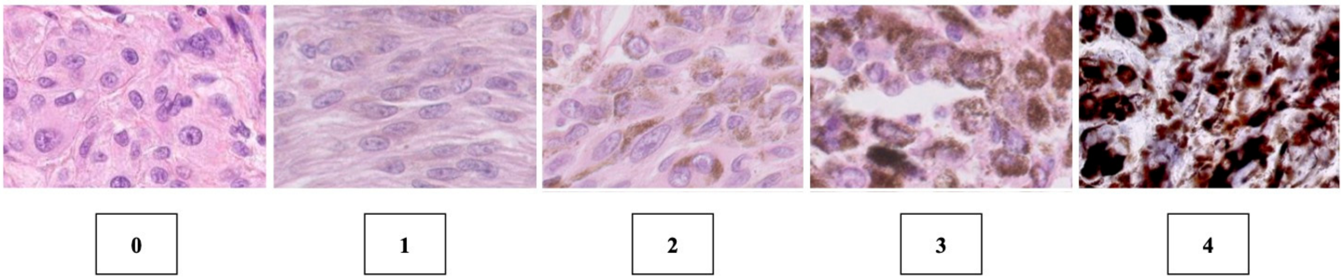

Figure S3. Immunohistochemical assessment of pigmentation of liver metastases. The average intensity of cytoplasmic melanin in all melanoma cells in each metastasis with five-point scale was based on examination of representative hematoxylin, eosin, saffron-stained sections. 0—absent, 1—faint, 2—moderate, 3—high, 4—very high.

References

1. Viros, A.; Fridlyand, J.; Bauer, J.; Lasithiotakis, K.; Garbe, C.; Pinkel, D.; Bastian, B.C. Improving Melanoma Classification by Integrating Genetic and Morphologic Features. *PLoS Med.* **2008**, *5*, e120, doi:10.1371/journal.pmed.0050120.
2. Barnhill, R.L. Institut Curie, Paris, France Unpublished work, 2021.
